# Characterizing Behaviors Associated with Enteric Pathogen Exposure among Infants in Rural Ecuador through Structured Observations

**DOI:** 10.4269/ajtmh.21-1099

**Published:** 2022-04-11

**Authors:** Andrea Sosa-Moreno, Gwenyth O. Lee, Amanda Van Engen, Kelly Sun, Jessica Uruchima, Laura H. Kwong, Elizabeth Ludwig-Borycz, Bethany A. Caruso, William Cevallos, Karen Levy, Joseph N. S. Eisenberg

**Affiliations:** ^1^ School of Public Health, University of Michigan, Ann Arbor, Michigan;; ^2^ School of Public Health, University of California Berkeley, Berkeley, California;; ^3^ Hubert Department of Global Health, Rollins School of Public Health, Emory University, Atlanta, Georgia;; ^4^ Instituto de Biomedicina Universidad Central, Quito, Ecuador;; ^5^ Department of Environmental and Occupational Health Sciences, University of Washington, Seattle, Washington

## Abstract

The relative importance of environmental pathways that results in enteropathogen transmission may vary by context. However, measurement of contact events between individuals and the environment remains a challenge, especially for infants and young children who may use their mouth and hands to explore their environment. Using a mixed-method approach, we combined 1) semistructured observations to characterize key behaviors associated with enteric pathogen exposure and 2) structured observations using Livetrak, a customized software application, to quantify the frequency and duration of contacts events among infants in rural Ecuador. After developing and iteratively piloting the structured observation instrument, we loaded the final list of prompts onto a LiveTrak pallet to assess environmental exposures of 6-month infants (*N* = 19) enrolled in a prospective cohort study of diarrheal disease. Here we provide a detailed account of the lessons learned. For example, in our field site, 1) most mothers reported washing their hands after diaper changes (14/18, 77.8%); however only a third (4/11, 36.4%) were observed washing their hands; 2) the observers noted that animal ownership differed from observed animal exposure because animals owned by neighboring households were reported during the observation; and 3) using Livetrak, we found that infants frequently mouthed their hands (median = 1.9 episodes/hour, median duration: 1.6 min) and mouthed surroundings objects (1.8 episodes/hour, 1.9 min). Structured observations that track events in real time, can complement environmental sampling, quantitative survey data and qualitative interviews. Customizing these observations enabled us to quantify enteric exposures most relevant to our rural Ecuadorian context.

## INTRODUCTION

Enteric pathogens are transmitted through pathways such as ingestion of contaminated water or food or through oral contact with hands, fomites, and flies.^1^ As a result, unsafe feeding and hygiene practices are widely associated with a higher risk of enteric infections, especially in resource-limited communities with abundant fecal exposure due to poor sanitary conditions.^2^^–^^4^ Many enteric pathogens cause diarrhea, which is one of the top five causes of morbidity and mortality in children under age 5, and also undermine nutrition, growth, and cognitive development.^5^^,^^6^ There is wide recognition of the need to quantify the relative importance of environmental pathways and sources of enteric pathogen exposure.^7^ Recent studies have aimed to fill that gap by quantifying the contribution of each pathway to ingestion of fecal indicator bacteria.^8^^,^^9^

Context-specific instruments to explore environmental exposure pathways that result in transmission have been developed and applied to various populations, especially for infants and young children who use their mouth and hands as tools to explore their environment.^10^^,^^11^ These instruments can overcome some of the issues associated with self-reported questionnaires, such as over-report of desirable behaviors and recall bias. To overcome those limitations, trained observers can be used to record a subject’s behavior. Observational methods that quantify infant-environment interactions have been used in water, sanitation, and hygiene (WASH) research.^12^^–^^15^

Observations can be unstructured, semistructured, or structured. In unstructured observations the observer records all the participant’s activities without a predetermined plan.^16^ Unstructured observations are helpful in the initial stages of research because of their flexibility to capture information about complex topics. Semistructured observations focus on pre-established activities while also capturing other non-planned actions. Structured observations aim to characterize a fixed set of activities of interest to the researcher. Semistructured and structured observations have been used to describe water, sanitation, and hygiene (WASH) behaviors, such as handwashing,^17^^,^^18^ defecation and stool disposal,^12^^,^^16^^,^^19^ infant geophagy,^17^^,^^20^ or contact with animals and animal feces.^21^^,^^22^

Spot checks are another widely used observational method used for assessing a predetermined list of conditions.^23^^–^^27^ Spot checks can be used to evaluate inadequate environmental conditions surrounding infants, such as the absence of sanitation facilities,^28^ poor household cleanliness (presence of feces on the floor, stagnant wastewater),^23^^,^^28^^–^^30^ and hand cleanliness,^27^^,^^31^ all of which are associated with enteric outcomes. Hour-to-hour spot check results can estimate the short-term variability of certain behaviors. Activities that vary rapidly over short periods of time are better captured in repeat measurements. However, both structured observations and spot checks may require repetition over longer periods to capture long-term change over time.

Structured observations have been used to characterize oral contact behaviors, defined by Davis, et al. as “oral contact with any object, surface, liquid, or body part (own or other)”.^15^ Contact rates between individuals has been disaggregated by infant age,^15^^,^^32^ developmental stage,^33^ or maternal or family behaviors, among others. Researchers have used structured observation instruments developed with electronic survey software such as Open Data Kit (ODK)^15^^,^^32^ or videography^34^ to capture frequency of episodes or individual contacts. An additional tool used for structured observations is the Android app LiveTrak (Stanford University, CA; open-source link: https://github.com/chrisdembia/LiveTrak). This app enables observers to use a customizable pallet to track the initiation and duration of events in real time and has been used to characterize events that may increase risks of enteric pathogen exposure.^13^ The pallet appears on the Android tablet screen as a grid of buttons that can be selected by the observer to indicate that certain behaviors are ongoing. For example, the observers would select a button to indicate the presence of an animal in the same space as the child. The LiveTrak software application may increase the accuracy of characterizing infant’s exposures to environmental hazards by capturing detailed information about the frequency and duration of pre-specified behaviors.^35^ The ability to customize the LiveTrak pallet is also desirable given that WASH behaviors and oral contacts may vary widely between populations and settings.^32^

This is a hypothesis-generating study where we describe an approach to assemble a prespecified list of actions potentially associated with infant environmental exposure (“the instrument”) through semistructured observation and then implement this instrument as a customized LiveTrak pallet to 1) record details of behaviors associated with enteric pathogen exposure (handwashing, diaper changing, and feeding) and 2) quantify the frequency and duration of contact episodes among infants in the province of Esmeraldas, Ecuador.

## METHODS

### Study setting and population.

The Gut Microbiome, Enteric Infections, and Child Growth across a Rural–Urban Gradient or Enteropatogenos, Crecimiento, Microbioma y Diarrea (EcoMID) study is a prospective cohort study of infants living in northern Ecuador from birth to age 2 years.^36^ One of the study’s goals is to comprehensively characterize environmental enteropathogen exposures affecting young children through a combination of environmental microbiology, qualitative, survey-based, and structured observations methods.

To inform the development of the structured observation instrument used in EcoMID, we conducted a pilot study from June to August of 2018 in Esmeraldas (population ∼200.000), a coastal city in northwestern Ecuador. For the pilot study we enrolled mother–infant dyads in which the mother was aged 18 years or older, self-identified as Afro-Ecuadorian, and had an infant aged 0 to 2 years with no known severe developmental delays or severe health issues (such as congenital heart disease or cerebral palsy). We recruited participants by convenience sample among mother–infant dyads attending child wellness visits at the district health center and participant referrals. After the pilot study, the EcoMID birth cohort began enrollment in May 2019 in the city of Esmeraldas, the town of Borbón (population ∼6000), and the small towns of Maldonado (population ∼1.500) and Zancudo (population ∼368). The EcoMID cohort study enrolled women in late pregnancy who were aged 18 years or older, regardless of Afro-Ecuadorian or mestizo ethnicity. Initially, all infants recruited in EcoMID were to participate in the structured observation study. However, only the first 19 infants who turned 6 months were able to participate in the observations before the activity was paused due to the COVID-19 pandemic. No sample size calculation was performed. Five observers participated in Phase 1 and Phase 2, whereas in Phase 3, two of the original observers and two new observers participated.

We obtained written informed consent from the mothers in Spanish, the local language, for both the pilot study and the cohort study. The protocol for this pilot study was approved by ethical review boards at the University of Michigan in the U.S. (HUM00142759) and the Universidad San Francisco de Quito in Ecuador (2011_02). The protocol for the parent EcoMID study was approved by ethical review boards at Emory University in the U.S. (IRB00101202) and the Universidad San Francisco de Quito in Ecuador (2018-022M).

### Instrument development.

**Figure 1.**
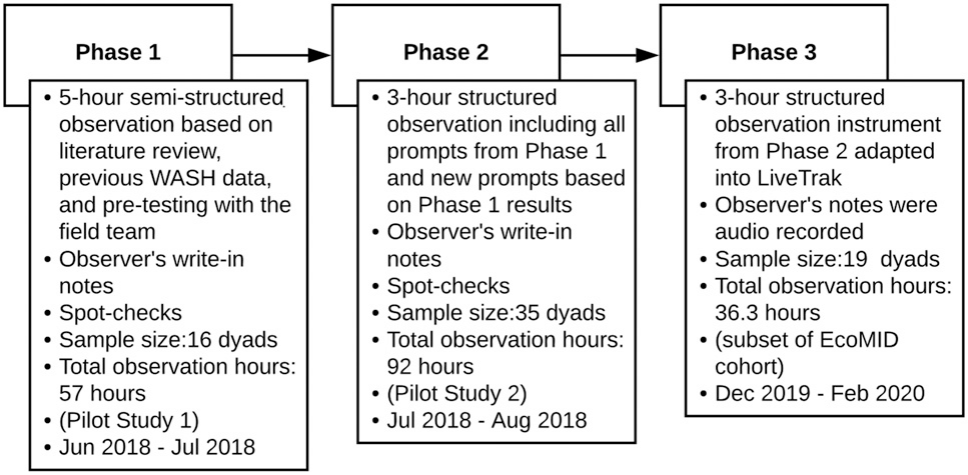
Flow chart of study phases. EcoMID = Enteropatogenos, Crecimiento, Microbioma y Diarrea; WASH = water, sanitation, and hygiene behaviors.

We developed the items that made up our structured observation instrument through an iterative three-phase process. In Phase 1, we identified site-specific activities known to be important sources of enteropathogen exposure and other infant–environment interactions. We captured those activities using a semistructured observation instrument. In Phase 2, we adjusted prompts from Phase 1 into a shorter structured observation instrument. In Phase 3, we adapted prompts from Phase 2 and programmed them into LiveTrak, allowing us to quantify exposures in real time. Additionally, we conducted repeated spot checks in Phase 1 and 2 to quantify the hour-to-hour variation of household and hand cleanliness conditions throughout the observation time. The results allowed us to decide how often a behavior would be measured in subsequent EcoMID questionnaires: once per questionnaire (for behaviors with low hour-to-hour variation) or over three separate visits (for behaviors with high hour-to-hour variation). The three phases are shown in Figure 1 and detailed in what follows.

#### Phase 1 (Pilot Study 1).

To identify site-specific activities in our study sites known to be important sources of enteric pathogen exposure elsewhere, we developed the first version of a 5-hour semistructured observation instrument through a combination of a literature review, an examination of WASH data from prior quantitative surveys conducted in the same region of Ecuador, and pretesting with the field team. The list of actions recorded included infant and maternal handwashing, diaper changing, and infant feeding, among others (see Supplemental Table 1 for a detailed description). Observers collected the data on paper forms. The observation was “semistructured” because it included a space for write-in responses and notes where observers detailed the events, including information on any other relevant activity not mentioned in the initial list and that would have been missed otherwise. Observers were trained to write notes at least every hour throughout the observation period. We defined mouthing as a single contact of the infant’s mouth with hands or objects, excluding food. We defined touching as a single contact of the infant’s hands with objects or people. We also conducted spot checks to assess hygiene conditions near the infant (e.g., hands visibly dirty, presence of stagnant water indoors and outdoors, presence of uncovered food and spilled food on the kitchen floor, and presence of feces in the home or yard). Spot checks were conducted upon arrival and every 60 minutes during the observation period.

#### Phase 2 (Pilot Study 2).

Results from Phase 1 informed the development of Phase 2 protocols. The observer’s notes and write-in responses in Phase 1 revealed that many infants napped in the second half of the observation (further described in the ‘Instrument Implementation’ section). Therefore, we adjusted prompts from Phase 1 into a shorter, 3-hour structured observation instrument that was also paper-based. The instrument included all prompts in Phase 1 and new prompts based on the Phase 1 results (see Supplemental Table 2 for a detailed description of activities). In contrast with Phase 1, in Phase 2 we recorded repeated instances of infant touching and mouthing as ‘episodes’ (repeated contacts that occurred within five seconds of each other) rather than as distinct contacts. During piloting and training, we found that episodes were easier for observers to recognize compared with individual contacts.

#### Phase 3 (subset of EcoMID cohort).

**Figure 2.**
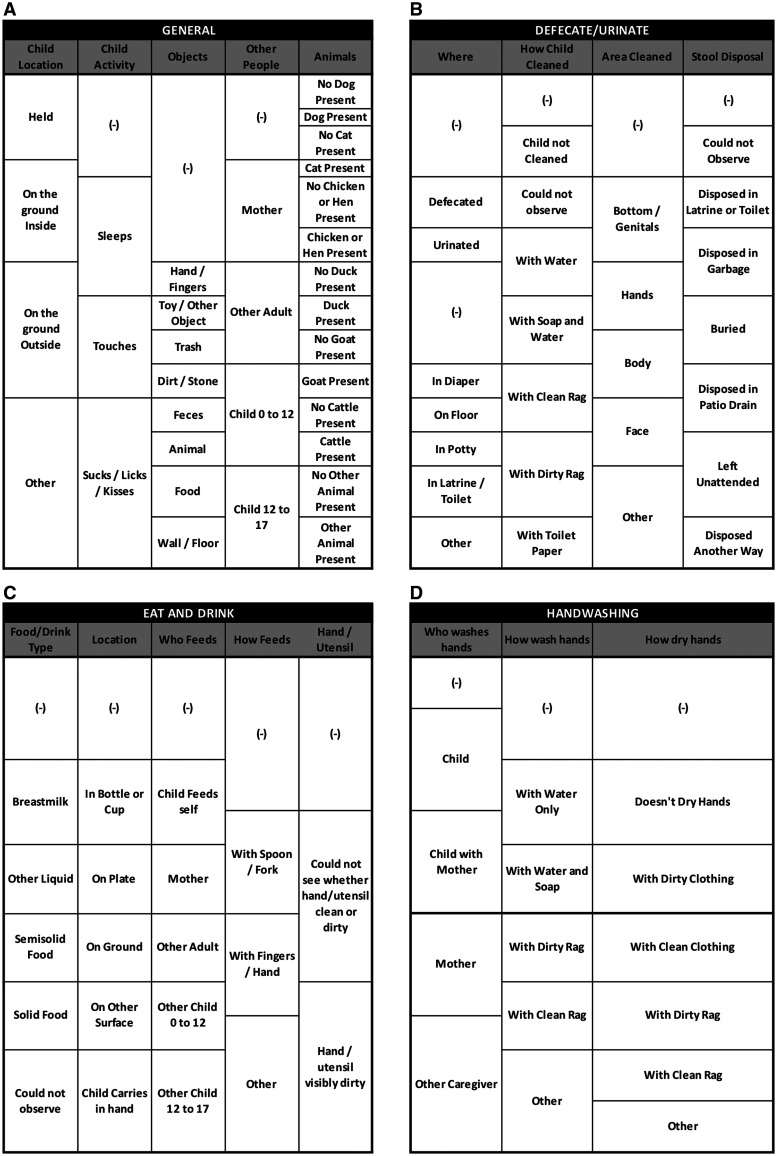
The customized structured observation pallet in LiveTrak (Phase 3).

We incorporated the results from Phase 2 into the LiveTrak app for real time, paperless data collection. Each activity was represented by a button in the LiveTrak pallet, so observers were able to click when a behavior started. The customized structured observation pallet in LiveTrak (Phase 3) is shown in Figure 2. To register the end of the activity, the observer could either click on a blank button if no other activity followed or click on another button corresponding to a different behavior. The instrument recorded the infant’s location (held or on someone’s lap/on the ground indoors/on the ground outdoors/other); whether the infant was sleeping; the presence of animals; and handwashing, feeding, and diaper change behaviors. Consistent with Phase 2, we recorded mouthing and touching of hands, toys/other objects, trash, dirt/stone, feces, animals, food, wall/floor as episodes (rather than distinct contacts). Handwritten notes were replaced with audio recordings embedded in an Open Data Kit (ODK) survey for observers to dictate any final observations or notes at the close of each visit. We implemented Phase 3 in the cohort by observing each participating infant when they turned 6 months old (±2 weeks). This activity was conducted from December 2019 to February 2020.

### Instrument implementation.

In all phases, trained personnel from the same communities as the participants carried out the observations. All observations were conducted by female observers, except for one observation in Phase 3. Female observers were favored to make mothers and their infants feel more comfortable during the observation period. Observers were trained to record behaviors while sitting quietly and observing, without interacting with the household members unless the infant was in imminent danger. For Phase 1 and 2 trainings, two observers completed an observation of the same infant concurrently and then compared results and discussed discrepancies. For Phase 3 training, observers used publicly available videos of noncohort children playing while capturing their activities using LiveTrak.

During all phases, observers scheduled observations to start when the infant typically woke up in the morning or after they woke up from a nap (mean time 10:30 am). We chose this approach to increase the likelihood that the field team would capture the child awake and observe at least one diaper change and one feeding episode. One observer was assigned per observation. Observers noted caregiver behaviors only when they occurred during an interaction with the infant—for example, immediately before, during, or after a diaper changing event.

### Additional variables.

We also collected basic sociodemographic data as well as characteristics that have previously been associated with infant–environment interaction.^32^ These includes: age, sex, and mobility of the infants; the age, marital status, and parity of the mother; animal ownership, type of sanitation facility and the community where the household was located. These variables were collected using a questionnaire administered to the caregiver.

### Analysis.

The frequency of episodes per hour was calculated for all the phases, whereas the duration of episodes was calculated only for Phase 3. Throughout the results, we calculated median values of episodes to reduce the impact of a few extreme outliers. We excluded from the analysis the time that an infant was asleep or could not be observed—for instance, when the infant went into a neighbor’s home or a private part of the house where the observer could not follow. For the repeated spot checks, we calculated the mean and 95% confidence intervals for the intraclass correlation coefficient (ICC) to measure hour-to-hour variation of cleanliness conditions during the observation period.^37^ ICC values vary from 0 to 1.^38^ We selected the spot check results at all available time points during Phase 1 and Phase 2 to calculate the ICC. In Phase 3, all spot checks items were passed from the structured observation instrument to household questionnaires. For our analysis, items with an ICC value of 0.59 or higher were checked in one visit, whereas items with an ICC value lower than 0.59 were checked over three separate visits.^39^ We used Cohen’s kappa test to assess agreement between reported and observed household animal presence. All analyses were done with R version 3.4.1^40^ or Stata version 16.1.^41^

## RESULTS

### Subject and household characteristics.

In total, 16 mother–infant dyads participated during Phase 1, 35 dyads during Phase 2, and 19 dyads during Phase 3. Characteristics of the study population are available in Table 1. The average age of infants in the pilot studies was 10.5 months (range: 1 week–23.5 months), whereas all infants in Phase 3 were 6 months old.

**Table 1:** Descriptive characteristics of study participants from the pilot and the cohort study

	Phase 1 and 2 (*N* = 45)	Phase 3 (*N* = 19)
	*n* (%)	*n* (%)
Location		
Esmeraldas	45 (100)	9 (47.4)
Borbón	0	6 (31.6)
Maldonado	0	3 (15.8)
Zancudo	0	1 (5.3)
Observed child age (months)*	10.5 (6.1)	6 (–)
Male, *n* (%)	28 (37.8)	9 (47.4)
Child mobility		
Cannot yet sit with support	11 (24.4)	5 (29.4)†
Can sit with support	6 (13.3)	14 (82.4)†
Can sit without support	5 (11.1)	4 (23.5)†
Can crawl	1 (2.2)	8 (47.1)†
Can stand	1 (2.2)	10 (58.8)†
Can walk alone	5 (11.1)	0†
Can run	11 (24.4)	0†
Mother’s age, years*	25.3 (6.6)	25.2 (7.0)
Marital status		
Married/in a relationship	10 (66.7)	16 (84.2)
Divorced/separated	1 (2.2)	0%
Single/never married	14 (31.1)	3 (15.8)
Number of live births		
1	16 (35.6)	7 (36.8)
2	18 (40.0)	5 (26.3)
≥ 3	11 (24.4)	7 (36.8)
Any animals present in the household	16 (35.6)	11 (57.9)
Access to improved sanitation facility	43 (100)‡	16 (94)†

* Mean (SD) are shown for these categories.

† Missing data: data were only available for 17 infants.

‡ Missing data: data were only available for 43 infants; two mothers declined to answer.

Observers completed 70 total observations: sixteen 5-hour observations in Phase 1, thirty-five 3-hour observations in Phase 2, and nineteen 3-hour observations in Phase 3. Six dyads from Phase 1 were also observed in Phase 2. Observations occurred over 185.3 hours when children were awake: 57 hours during Phase 1, 92 hours during Phase 2, and 36.3 hours during Phase 3.

### Phase 1.

Because observations were scheduled to begin at the infant’s usual wake-up time, no infants slept during the first 3 hours of the observation. Observers’ notes showed that infants sometimes slept (12.5%) or could not be observed (6.2%) at least once in the final 2 hours of the observation; children slept during 18.8% of hours during the fourth hour of observation (3/16), and during 6.3% of hours during the fifth hour of observation (1/16).

We observed 17 diaper changes, including at least one diaper change in 75.0% (12/16) of the observations (Table 2). All diaper changes were observed in the first 3 hours of the observation. Additional details were collected in the observer’s notes, for example: “The girl defecated inside the house on a potty. The mother disposed the feces in the backyard sewer, washed the girl only with water as well as the basin. She did not wash her hands” (observation of a 7-month-old). Similarly, infants were fed during seven of the 16 observations, all in the first 3 hours.

**Table 2:** Characteristics of observed diaper change events

	Phase 1 and 2 (*N* = 47)	Phase 3 (*N* = 13)
	*n *(%)	*n* (%)
What was done with the fecal materials?		
No fecal materials present	4 (8.5)	1 (7.7)
Wrapped and thrown in trash	37 (78.7)	11 (84.6)
Buried or tossed in yard	5 (10.6)	–
Not able to observe	1 (2.1)	1 (7.7)
Caregiver washed hands after cleaning child?		
No	20 (42.6)	8 (61.5)
Yes		
Rinsed with water and soap	20 (42.6)	2 (15.4)
Rinsed with water only	3 (6.4)	1 (7.7)
Other	–	2 (15.4)
Unable to observe	4 (8.5)	–
Child cleaned after diaper change?		
No	1 (2.1)	1 (7.7)
Yes		
Soap and water	13 (27.7)	–
Rinsed with water only	8 (17.0)	1 (7.7)
Wiped with cloth/baby wipes only	22 (46.8)	10 (76.9)
Wiped with paper only	1 (2.1)	–
Unable to observe	2 (4.3)	1 (7.7)

A total of 60 diaper change events were observed, 17 during Phase 1, 30 during Phase 2, and 13 during Phase 3. Of 70 observations, 45 (64.3%) included at least one diaper change event.

Mouthing and touching were frequently observed. For example, one observer noted the following scene: “The baby put the mother’s cell phone in their mouth, then the baby carried around a pot in their hand and their mouth. The baby drank water off the floor, then they put a coin in their mouth, then they put a piece of cloth in their mouth, then they put their hands in their mouth” (10-month-old).

Observers reported that many infants touched or mouthed objects repeatedly within a period of several seconds or minutes, and it was difficult to record the number of individual contacts as they occurred very close together in time or were not visible because the child’s hand was blocking their mouth. Observers also noted multiple interactions with animals, including not only household animals but also those owned by neighboring households, for example: “A neighbor’s cat entered the home” (5-month-old infant), “A street cat came inside” (11-month-old infant), and “A cat that doesn’t belong to the household entered the house” (17-month-old).

Finally, fieldworker’s notes from Phase 1 captured behaviors not mentioned in the prespecified list of activities. For example, although kissing was not included in the initial version of the observation form, it was reported frequently in narrative observation notes. For instance, observers described the following scenes:

*The father kisses the baby’s mouth. The father kisses the baby’s cheek. … the mother kisses the baby on the mouth. The sister kisses the baby’s face twice. (7-month-old)*

*The sister touches the baby’s mouth, and then puts her nose in the baby’s mouth. The uncle sucks on the baby’s little fingers and kisses the baby, then the mother kisses the baby on the mouth. (10-month-old)*

### Phase 2.

On the basis of our experience in Phase 1, we decreased observation length to 3 hours to avoid time infants spend mostly sleeping.

As in Phase 1, we characterized diaper changes. We observed 30 diaper changes, including at least one diaper change in 22 of 35 (62.9%) observations (Table 2). Observers asked mothers whether they wash their hands after diaper changing events. Most mothers (32/35) reported that they had. However, the structured observation showed that only 50.0% (15/30) of observed events were followed by handwashing with soap and water. In other instances, mothers rinsed with water only (2/30), or washed only one hand (1/30), or did not wash in any way (11/30). Mothers most often cleaned their infants with baby wipes (60.0%), followed by soap and water (26.7%).

Defining a series of the same type of contacts close in time as a single “episode,” we recorded 70 of 92 (76.1%) observation hours with at least one instance of another person kissing the infant on the face and a median of two episodes per hour (interquartile range [IQR)]: 1–3). Infants frequently mouthed hands (71 of 92 hours with at least one episode): a median of 1 episode per hour was observed (IQR: 1–4) (Table 3). Infants were also observed touching other people, animals, walls, or ground and making frequent oral contact with toys, trash, dirt/stones, food, clothing objects such as sandals and watches, and household objects such as plastic bags, tin cans, paper, and plastic containers. Interaction between infants and any animal was noted during 10 of 92 hours of observation. In Phase 2, observed animals were dogs (seven observations), cats (four observations), and chickens (two observations). Infants interacted with animals during four of 32 total observations (8/92 hours) during Phase 2. In 10 instances, the family reported animal ownership and no animals were observed, and in one instance, the family reported no animal ownership but animals were observed. Cohen’s kappa was 0.20 (agreement due to chance: 57%, observed agreement, 66%).

**Table 3:** Frequency of contact behaviors: mouthing, touching, and kissing

Action	Phase	Proportion of hours with an oral contact episode	Median oral contact events/hour (25th, 75th percentile)	Mean oral contact events/hour (SD)
Mouthing hands	2	71/92 (77.2%)	1 (1, 4)	2.5 (2.9)
	3	–	1.9 (0.7, 2.7)	2.8 (2.3)
Mouthing outside objects like rocks or garbage from the street	2	16/92 (17.4%)	0 (0, 0)	0.37 (1.1)
Mouthing indoor objects like toys, kitchen containers, etc.	1 and 2	53/92 (57.6%)	1 (0, 1)	0.58 (0.5)
	3	–	1.8 (0.7, 2.6)	2.1 (2.5)
Touching objects like toys	3	–	2.5 (1.6, 5.1)	4.6 (5.6)
Infants being kissed by others	2	70/92 (76.1%)	2 (1, 3)	2.1 (2.0)
	3	–	1.4 (0.4, 2.7)	2.0 (2.2)

The prevalence of observing dirt on hands during spot checks in Phase 1 and 2 was 17.2% among mothers/caregivers and 33.3% among infants. However, both measures showed high hour-to-hour variability with ICCs of 0.42 and 0.55, respectively. Other spot check ICCs such as “uncovered food” and “kitchen spills” showed low hour-to-hour variation throughout the observation, with ICCs higher than 0.9 (Table 4).

**Table 4:** Spot check results and ICCs for hour-to-hour variability

	Phase	% Of unobservable observations	Prevalence	ICC
Caregiver hands dirty	1 and 2	33/149 (22.2%)	20/116 (17.2%)	0.42 (0.14, 0.77)
Child hands dirty	1 and 2	17/149 (11.4%)	44/132 (33.3%)	0.55 (0.27, 0.80)
Stagnant water visible indoors	2	4/92 (4.4%)	25/88 (28.4%)	–
Stagnant water visible outside	2	1/92 (1.1%)	10/91 (11.0%)	–
Stagnant water visible inside or outside	1 and 2	11/149 (7.4%)	61/138 (44.2%)	–
Unwashed utensils or cookware	1 and 2	26/149 (17.5%)	67/123 (54.5%)	0.74 (0.46, 0.90)
Uncovered food that is not being eaten	1 and 2	33/149 (22.2%)	24/116 (20.7%)	0.92 (0.30, 1.00)
Spill on kitchen floor	1 and 2	27/149 (18.1%)	36/122 (26.2%)	0.98 (0.88,1.00)
Visible feces in home or yard	1 and 2	9/149 (6.0%)	14/140 (10.0%)	0.72 (0.26, 0.95)
Dusty kitchen and dining area	1 and 2	12/149 (8.1%)	55/137 (40.1%)	0.88 (0.52, 0.98)
Cleanliness of the area where the child is located*	1 and 2	51/149 (34.2%)	0.97 (1.29)†	0.88 (0.79, 0.93)

ICC = intraclass correlation coefficient. Spot checks are observations of a predetermined list of conditions at defined times.

* Percent of time not applicable; 0 = not clean, 5 = very clean.

† Mean (SD).

### Phase 3.

**Figure 3.**
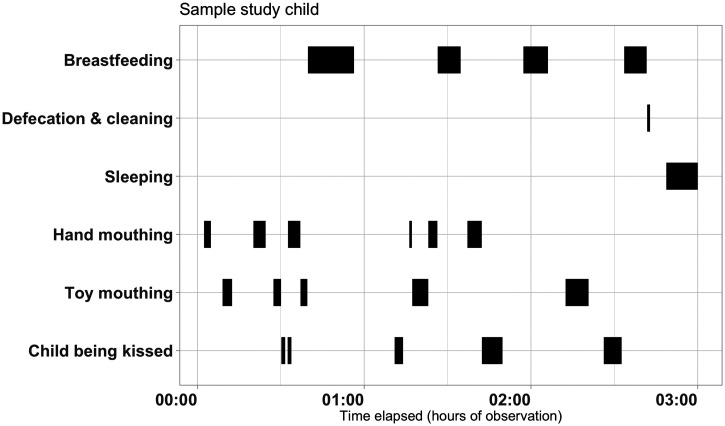
Visual depiction of the data resulting from observing an infant using the customized structured observation pallet in LiveTrak.

We customized the LiveTrak pallet based on the Phase 2 prompts. The customized pallet included four tabs to capture information on distinct topics: General (infant’s location and behaviors such as mouthing and touching), defecate/urinate (waste disposal and after cleaning), eat/drink (type of food and use of utensils), and handwashing (hand cleaning) (Figure 2). A visual depiction of the data resulting from this instrument for one infant is shown in Figure 3. We observed 13 diaper changes during Phase 3 in 57.9% (11/19) of the observations (Table 2). We found a median of 1.9 and 1.8 hand-mouthing and object-mouthing episodes per hour, respectively (Table 3). The median hand-mouthing episode lasted 1.6 min (IQR: 0.9–3.8 min), while the median duration of mouthing objects was 1.6 min (IQR: 0.2-3.7 min). In Phase 3, 75.0% of mothers reported washing hands after diaper changes (14/18, 77.8%), whereas 4 of 11 (36.4%) were observed washing their hands after a changing episode. Most mothers observed in Phase 3 cleaned their infant with baby wipes (8/11, 72.7%). Most infants who defecated in Phase 3 were cleaned afterward (10/13, average cleaning time: 84 seconds).

Animals were observed during the observation period: dogs (two observations), chickens (two observations), cats (five observations), and a duck (one observation). However, only two infants were observed touching animals. Touching episodes lasted 174 and 55 seconds, respectively.

Observers used audio recordings to provide additional details about activities and about unusual aspects of the observation. The following is an observer’s note about an infant who was held by her mother throughout the observation:

*They don’t put the child on the floor, they just hold her in their arms. The child was weak … because she got her vaccine. That’s why they did that. (6-month-old)*

## DISCUSSION

Infancy is a period of rapid motor development, accompanied by intense engagement and exploration of the environment that may increase the risk of enteric pathogen exposure. Oral contacts vary over time in frequency and form,^13^ and vary among households depending upon their infant care practices. Quantifying these infant-environment interactions can help inform our understanding of how and why some children are more exposed to enteric pathogens than others and provide information on the relative importance of environmental transmission pathways compared with other pathways (e.g., food, water). In this paper we have described a methodological approach to customize a field instrument through semi-structured observation, and then implement the customized instrument to record key activities associated with potential exposure to enteric pathogens among infants in northern Ecuador.

Mouthing and touching of hands, play objects, surfaces, other people, and animals may be important opportunities for microbial exchange between infants and their environment and may be more critical transmission pathways for enteropathogens than drinking water.^8^^,^^15^^,^^30^^,^^42^ Similar to other studies using structured observations, our results show that mouthing and touching episodes were frequent among infants.^15^ The implementation of the structured observation instrument using the LiveTrak app allowed us to record duration of episodes while tailoring the instrument to the local context, providing the potential to better characterize environmental exposure for cohort children. In addition, we were able to record activities that are less accurately reported via questionnaires. For example, participants tend to overreport desirable behaviors such as handwashing and fecal management and disposal.^12^ A participant’s ability to accurately remember behaviors could also introduce recall bias.^43^^,^^44^

Some of our findings differed from those in similar studies. Infant contact with animals and animal feces was less common than prior studies in rural communities in other parts of the world. Although we reported animals entering the household, we did not record any interaction between infants and animal feces during the observation time. Marquis, et al., reported 3.9 feces-to-mouth episodes per 12 hours per infant.^45^ A study in rural Zimbabwe, found 2 instances of infants consuming chicken feces among 130 observation hours.^17^ During Phase 1, we also noted that infants were frequently kissed on the face. Kissing is not thought to be a risk factor for enteric pathogen transmission, although it is sometimes discouraged by pediatricians as a risk for respiratory disease, especially for newborns.^46^^,^^47^ However, it is also a form of close contact between adults, older children, and infants that is positive for social and psychological development.^48^^–^^50^ Given that kissing was common, we decided to continue to capture this behavior in the LiveTrak pallet for further evaluation.

Our results also have several methodological implications for future observation-based research of infant oral contact or hygiene behaviors. First, there is variability in the length of observations among the literature: some studies have used 2.5- or 3-hour observations,^51^^,^^52^ others 5-hour observation or more.^13^^,^^17^^,^^30^^,^^32^ We found that a 3-hour observation (Phase 2 and 3) was less tiring for both observers and participants than a 5-hour observation (Phase 1). Shortening the observation time allowed us to avoid time infants spend mostly sleeping. Second, scheduling the visit in advance allowed us to assess at least one feeding event and one diaper changing event in most instances. Third, hourly spot check revealed high temporal variability of some conditions. Had only one spot check per day been conducted to observe these contexts, we could have introduced misclassification bias. Conditions with the greatest temporal variability may be best suited to structured observation, whereas conditions with moderate variability may be spot-checked several times to obtain a measure of the “typical” condition, and conditions with the lowest variability may be spot-checked less often. For example, highly variable conditions such as observing dirt on the infants hands would need to be captured repeatedly to assess “typical” exposure. However, spot checks of items such as “uncovered food,” “visible food,” and “kitchen spills” were relatively stable across the observation and may be reliably integrated into short surveys. Finally, observer’s notes and write-in responses during Phase 1 and 2 recorded activities not mentioned in the initial list. These notes were used to customize the observation instrument into a new environment. Unstructured notes were also useful in later phases, after formative research, in understanding the context of some activities.

Our study also has several limitations. First, we intended for our observation instrument to quantify the frequency and duration of oral contacts. However, given the difficulty our staff experienced in distinguishing discreet oral contact events during mouthing, we chose to record “episodes” (repeated instances of mouthing in a short period), rather than each individual contacts. As a result, we report a similar frequency compared with episode-based studies^15^ and a much lower frequency than studies where contacts were captured individually.^13^^,^^32^ For example, we reported a hand-mouthing median frequency of 1.9 episodes per hour, Davis et al., reported 0.4 episodes per hour, and Kwong et al. reported a frequency of 43 contacts per hour. Similarly, we reported an object-mouthing median frequency of 1.8 episodes per hour, Davis et al. reported 0.4 episodes per hour, and Kwong et al. reported 34 contacts per hour.^13^^,^^15^ Individual contacts can always be summarized into episodes in subsequent analysis, but the reverse is not possible. Future work should compare these two metrics’ correlation to determine whether episodes are appropriate proxies for individual contact events. Second, we focused our study on observing mainly infants, caregivers were observed only when there was an interaction with the infants. Therefore, observers could have missed maternal/caregiver activities, such as handwashing events before and after food preparation, which may be a relevant factor for infant enteropathogen exposure. Third, our study aimed at observing infant behaviors that may expose them to enteropathogens. However, an assessment of pathogen loads along the identified pathways is needed to understand the relative importance of enteropathogen transmission pathways. Fourth, although we held training sessions for observers during which discrepancies in coding were identified and discussed, we lack interrater reliability data under real-world conditions. Finally, although we were able to learn from the implementation of the different phases of our study, our conclusions regarding exposure behaviors rates are based on a limited sample size and therefore should be interpreted with caution. Future studies aimed at characterizing exposure behaviors using structured observations should use larger sample sizes.

A phased process of instrument development, focusing initially on unstructured observer’s notes, allowed us to capture high-risk activities that would otherwise have been missed. The customizable LiveTrak application further enabled us to quantify the frequency and duration of oral contact episodes: a key component in enteric pathogen transmission. This integration of qualitative (observer’s notes) and quantitative methods (structured observations using LiveTrak) supports the quantification of enteric exposures most relevant to our rural Ecuadorian context and the development of contextually sufficient interventions to reduce those exposures among infants.

## Supplemental Material


Supplemental materials
