# Supplementary material for: Characterizing Behaviors Associated with Enteric Pathogen Exposure among Infants in Rural Ecuador through Structured Observations
Source: Am J Trop Med Hyg. 2022 Apr 11;106(6):1747–56. doi: 10.4269/ajtmh.21-1099 (PMC9209906; doi:10.4269/ajtmh.21-1099)
Supplement: Supplementary file 1 [file tpmd211099.SD1.pdf]

**Supplemental Table 1:** Semi-structured instrument used in Phase 1

| STRUCTURED OBSERVATION FORM | | | |
|---|---|---|---|
| # | Question | Code | Response |
| 1 | Maternal ID | FW number + M + household number | ☐ ☐ ☐ ☐ ☐ ☐ |
| 2 | Date | DAY / MONTH / YEAR | ☐ ☐/☐ ☐ ☐ / ☐ ☐ |
| 3 | Fieldworker ID | Fieldworker initials | ☐ ☐ |

| Infant Development Observation | | | |
|---|---|---|---|
| 1 | Motor development: Observe and check all that apply | ☐ a. Laying, no sitting or movement<br>☐ b. Sitting with support<br>☐ c. Sitting without support<br>☐ d. Creep on stomach<br>☐ e. hands-and-knees crawing | ☐ f. standing with assistance<br>☐ g. standing along<br>☐ h. walking alone<br>☐ i. run<br>☐ j. other (please note) |
| 2 | Behavior & health: Observe and check all that apply | ☐ a. Infant appears healthy. (Infant is alert and active when awake, is feeding well, and can be comforted when crying)<br>☐ b. Infant is lethargic. (Has little or no energy, drowsy or sluggish, difficult to wake for feedings, Not alert or attentive to sounds and visual stimulation)<br>☐ c. Infant is persistently crying or irritable (Continuously fretful and fussy, Cries for long periods or very suddenly, Has a cry that sounds unusual)<br>☐ d. Infant seems sick (is pale or flushed, has problems breathing, is vomiting or has diarrhea)<br>☐ other (please note): | |

| Child Behavioral Ratings | | | | | | | | |
|---|---|---|---|---|---|---|---|---|
| | **Question** | **Responses** | **On arrival** | **Hour 1** | **Hour 2** | **Hour 3** | **Hour 4** | **Hour 5** |
| | Child positive mood | 1 (unhappy)-5 (joyful) | | | | | | |
| | Child negative mood | 1 (content)-5 (constantly negative) | | | | | | |

| | | | | | | | | |
|---|---|---|---|---|---|---|---|---|
| | Lively/active | 1 (inactive)-5 (highly active) | | | | | | |
| | Sociability | 1(not social) -5 (highly social) | | | | | | |
| | Sustained Attention | 1 (distracted, inattentive)-5 (child is focused and involved) | | | | | | |
| | Demandingness | 1 (communicates needs appropriately) -5 (crying and tantrums for attention) | | | | | | |
| **Hourly Observations** | | | | | | | | |
| | **Question** | **Responses** | **On arrival** | **Hour 1** | **Hou r 2** | **Ho ur 3** | **Hour 4** | **Hour 5** |
| | Caregiver's hands visibly clean? | No=00; Yes=01; Cannot see=09 | | | | | | |
| | Baby's hands visibly clean? | No=00; Yes=01; Cannot see=09 | | | | | | |
| | Diaper or child's bottom is clean? | No=00; Yes=01; Cannot see=09 | | | | | | |
| | There is stagnant water visible? | No=00; Yes=01; Cannot see=09 | | | | | | |
| | There are unwashed utensils? | No=00; Yes=01; Cannot see=09 | | | | | | |
| | There is uncovered food that is not being eaten? | No=00; Yes=01; Cannot see=09 | | | | | | |
| | Spill on kitchen floor (food or drink) | No=00; Yes=01; Cannot see=09 | | | | | | |
| | There are poultry feces visible on kitchen floor? | No=00; Yes=01; Cannot see=09 | | | | | | |
| | There are animals in the house? | No=00; Yes=01; Cannot see=09 | | | | | | |
| | If yes, list animal types & number | e.g. 2 goats, 3 chickens | | | | | | |
| | Kitchen yard is swept | No=00; Yes=01; Cannot see=09 | | | | | | |

| | | | | | | | | |
|---|---|---|---|---|---|---|---|---|
| | Where is the child at this time? Dirt floor inside=01; finished floor inside=02; dirt floor outside=03; finished floor outside=04; on mother (hip)=05; on mother (lap)=06, on mother (back)=07; on mat or blanket=08; Other (specify)=09 | | | | | | | |
| | How many chickens are within 5 meters of meters? | Number of chickens | | | | | | |
| | Are feces visible within 5 meters of the child? | No=00; Yes=01; Cannot see=02 | | | | | | |
| | Is the area where the child is playing swept/clean? | No=00; Yes=01; Cannot see=02; Not applicable=04 | | | | | | |
| **Number of animals present in yard at time of observation** | | | | | | | | |
| | **Question** | **Responses** | **On arrival** | **Hour 1** | **Hou r 2** | **Ho ur 3** | **Hour 4** | **Hour 5** |
| | Cattle - corralled | Number of animals | | | | | | |
| | Cattle - not corralled | Number of animals | | | | | | |
| | Goats - corralled | Number of animals | | | | | | |
| | Goats - not corralled | Number of animals | | | | | | |
| | Chickens- corralled | Number of animals | | | | | | |
| | Chickens - not corralled | Number of animals | | | | | | |
| | Ducks - corralled | Number of animals | | | | | | |
| | Ducks - not corralled | Number of animals | | | | | | |
| | Dogs | Number of animals | | | | | | |
| | Cats | Number of animals | | | | | | |
| | Other animal 1: ______________________ | Number of animals | | | | | | |
| | Other animal 2: | Number of animals | | | | | | |

| | | | | | | | | |
|---|---|---|---|---|---|---|---|---|
| | ____________________<br>_ | | | | | | | |
| | Other animal 3:<br>____________________<br>_ | Number of animals | | | | | | |

| | | | | | | | |
|---|---|---|---|---|---|---|---|
| **Child Sanitation practices** | | | | | | | |
| | | **Diaper Changing / Cleaning Event #** | | | | | |
| | | **1** | **2** | **3** | **4** | **5** | **6** |
| | Time of Event, HH :MM; use miltary time | | | | | | |
| | Who initated cleaning the child? Child indicated need=01; caregiver=02; not able to observe=09 | | | | | | |
| | What was done with the fecal materials? disposed of via toilet/latrine=01; wrapped up for disposal in garbage=02;buried=03; tossed in yard=03; left untended for >30 min=05; not able to observe=09 | | | | | | |
| | How was the child's bottom cleaned?<br>Soap and water=01; rinsed only with water=02; wiped with cloth only=03; wiped with paper=04; not able to observe=09 | | | | | | |
| | Did caregiver wash his/her hands within 5 min after cleaning the child’s bottom (nappy change)? (skip) SKIP if child does not use nappies none/no=00; one hand=01; two hands=02; water only/no rubbing agent=03; Soap=04; Ash=05; Other=06; Run to waste water=07; still water=08; unable to observe=09) | | | | | | |
| | Did caregiver wash his/her hands within 5 min after assisting a toddler to clean up after defecation?<br>none/no=00; one hand=01; two hands=02; water only/no rubbing agent=03; Soap=04; Ash=05; Other=06; Run to waste water=07; still water=08; unable to observe=09) | | | | | | |

| | | | | | | |
|---|---|---|---|---|---|---|
| **Behavior Checklist** | | | | | | |
| | **Behavior** | **Number of Times Behavior was Observed in this Hour Block (TALLY)** | | | | |
| | | **Hour<br>0 to 1** | **Hour<br>1 to 2** | **Hour<br>2 to 3** | **Hour<br>3 to 4** | **Hour<br>4 to 5** |
| | Baby put in fingers in mouth? | | | | | |

| | | | | | | |
|---|---|---|---|---|---|---|
| | | | | | | |
| | Baby put dirt in mouth? | | | | | |
| | Baby put visibly dirty object in mouth | | | | | |
| | Baby put not visibly dirty object in mouth | | | | | |
| | Baby touched an animal | | | | | |
| | Baby ate freshly cooked food | | | | | |
| | Baby ate leftover food that was not covered | | | | | |
| | Baby drank water | | | | | |
| | Baby's hands were washed without soap / cleaning agent | | | | | |
| | Baby's hands were washed with soap / cleaning agent | | | | | |
| | Mother's hands were washed without soap / cleaning agent | | | | | |
| | Mother's hands were washed wit soap / cleaning agent | | | | | |
| | Other behavior #1 : ___________________ | | | | | |
| | Other behavior #2 : ___________________ | | | | | |
| | Other behavior #3 : ___________________ | | | | | |
| | Other behavior #4 : ___________________ | | | | | |
| | Other behavior #5 : ___________________ | | | | | |

Researcher Field Notes: Please describe any other details of behaviors observed during your observation today, interactions or other interesting or relevant information.

**Supplemental Table 2:** Semi-structured instrument used in Phase 2

| STRUCTURED OBSERVATION FORM | | | |
|---|---|---|---|
| # | Question | Code | |
| 1 | Child ID | FW number + C + household number | ☐ ☐ ☐ ☐ ☐ ☐ |
| 2 | Date | DAY / MONTH / YEAR<br>e.g. 04/FEB/18 | ☐ ☐ / ☐ ☐ ☐ / ☐ ☐ |
| 3 | Fieldworker ID | Fieldworker ID | ☐ ☐ |
| 4 | Age of Child | Number of Months | ☐ |
| 5 | Start Time of Observation | HH:MM | ☐ ☐ ☐ ☐ |
| 6 | End Time of Observation | HH:MM | ☐ ☐ ☐ ☐ |
| **Infant Development Observation** | | | |
| 1 | Motor development: Observe and check all that apply throughout the 5 hour observation | c a. Laying, no sitting or movement<br>c b. Sitting with support<br>c c. Sitting without support<br>c d. Creeping on stomach<br>c e. hands-and-knees crawing | c f. standing with assistance<br>c g. standing alone<br>c h. walking alone<br>c i. running<br>c j. other (please note) |
| 2 | Behavior & health: Observe and check all that apply throughout the 5 hour observation | c a. Infant appears healthy.<br>c b. Infant is lethargic.<br>c c. Infant is persistently crying or irritable<br>c d. Infant seems sick<br>c e. Other (please note): | |
| 3 | What animals are present on the property anytime throughout the 5 hour observation? Coralled is when the animal is confined in an area with no way of escaping. | c a. corralled cattle<br>c b. **not** corralled cattle<br>c c. corralled goats<br>c d. **not** corralled goats<br>c e. coralled chickens/baby chicks/roosters<br>c f. **not** coralled chickens/baby chicks/roosters | c g. corralled ducks<br>c h. **not** corralled ducks<br>c i. dogs<br>c j. cats<br>c k. Other animal 1: ___________<br>c l. No animals belong to the property |

| | | |
|---|---|---|
| 4 | Does an animal enter or attempt to enter the house at least once? (do not include dogs or cats) | Please describe. |
| 5 | People who come in contact with baby | |

| | | | | | | |
|---|---|---|---|---|---|---|
| **Hourly Observations** | | | | | | |
| | **Question** | **Responses** | **On arrival** | **Hour 1** | **Hour 2** | **Hour 3** |
| 1 | Caregiver's hands visibly clean? | No=00; Yes=01; Cannot see=09 | | | | |
| 2 | Baby's hands visibly clean? | No=00; Yes=01; Cannot see=09 | | | | |
| 3 | Is the baby sleeping? Note the time or for how long | No=00; Yes=01; Cannot see=09 | | | | |
| 4 | Stagnant water visible **indoors**? E.g. buckets of water with or without lid | No=00; Yes, both covered and uncovered=01; Yes, covered only =02; Yes, uncovered only =03; Cannot see=09 | | | | |
| 5 | Stagnant water visible **outdoors**? E.g. large puddles of water or standing pool | No=00; Yes, both covered and uncovered=01; Yes, covered only=02; Yes, uncovered only =03; Cannot see=09 | | | | |
| 6 | Are there unwashed utensils or cookware? | No=00; Yes=01; Cannot see=09 | | | | |
| 7 | Is there uncovered food that is not being eaten? (do not include food being cooked or in process of being cooked) | No=00; Yes=01; Cannot see=09 | | | | |
| 8 | Spill on kitchen floor (food or drink)? | No=00; Yes=01; Cannot see=09 | | | | |
| 9 | Are there any type of feces visible in the | No=00; Yes=01; Cannot see=09 | | | | |

| | | | | | | |
|---|---|---|---|---|---|---|
| | home or yard? | | | | | |
| 10 | Is the kitchen and dining area swept? | No=00; Yes=01; Cannot see=09 | | | | |
| 11 | Where is the child at this time? Dirt floor inside=01; Tiled floor inside=02; Dirt floor outside=03; Tiled floor outside=04; On mother (hip or lap)=05; On bed=07; On mattress or sofa=08; On baby seat=10; Cement floor inside=11; Cement floor outside=12; Wood floor inside=13; Wood floor outside=14; Cannot see=09; Other=888 | | | | | |
| 12 | Is the area where the child is playing swept/clean? | No=00; Yes=01; Cannot see=02; A little clean=03, Mostly clean=04; Very clean=05; Not applicable/on top mother=06 | | | | |

**Child Sanitation practices**

| | | Diaper Changing / Cleaning Event # | | | |
|---|---|---|---|---|---|
| | **Question** | **1st Time** | **2nd Time** | **3rd Time** | **4th Time** |
| 1 | Time of Event, HH :MM; use miltary time | | | | |
| 2 | What type of event is this? Full bath=01; Half bath= 02; Diaper/clothing change= 03 | | | | |
| 3 | Did the child defecate? No=00; In diaper= 01; In potty=02; In underwear= 03; On floor=04; Other=05; In bathroom=06 | | | | |
| 4 | Who initiated cleaning the child? Child indicated need=01; caregiver (adult)=02; Caregiver (child under 12)=03; Caregiver (child 12-18 years old)=04; Not able to observe=09 | | | | |
| 5 | What was done with the fecal materials? No fecal materials were present=00; Disposed of via toilet/latrine=01; Wrapped up for disposal in garbage=02; Buried=03; Tossed in yard in a drain=04; Left untended for >30 min=05; Not able to observe=09 | | | | |
| 6 | How was the child's bottom cleaned?<br>Soap and water=01; Rinsed only with water=02; Wiped with cloth only=03; Wiped with toilet paper=04; Not able to observe=09 | | | | |
| 7 | Did caregiver clean his/her hands within 5 min after cleaning the child or changing their diaper? No=00; One hand=01; Two hands=02; Other=03; Unable to observe=09 | | | | |

**Behavior Checklist**

| | | Number of Times Behavior was Observed in this Hour Block (TALLY) | | | | | |
|---|---|---|---|---|---|---|---|
| | | **Hour 0 to 1** | | **Hour 1 to 2** | | **Hour 2 to 3** | |
| | **Behavior** | **single event** | **sustained for more than 5** | **single event** | **sustained for more than 5** | **single event** | **sustained for more than 5** |

| | | | | | | | |
|---|---|---|---|---|---|---|---|
| | | | **seconds** | | **seconds** | | **seconds** |
| 1 | Baby put fingers in mouth | | | | | | |
| 2 | Baby puts outside objects in mouth like rocks or garbage from the street | | | | | | |
| 4 | Baby touched an animal | | | | | | |
| 3 | Baby walks or crawls out of the house on their own | | | | | | |
| 5 | Animal enters area with baby. Animal Type:________________ | | | | | | |
| 7 | Baby breastfed | | | | | | |
| 8 | Baby ate food | | | | | | |
| 9 | Baby ate food previously dropped | | | | | | |
| 10 | Baby drank water, juice or other drink | | | | | | |
| 11 | Baby's hands were washed **without** soap | | | | | | |
| 12 | Baby's hands were washed **with** soap | | | | | | |
| 13 | Baby's hands were washed (**unclear** with or without soap) | | | | | | |
| 14 | Others kiss or touch baby on the face | | | | | | |
| 15 | Baby initiates touching other people | | | | | | |
| 16 | Baby touched wall or ground with fingers | | | | | | |
| 17 | Baby is on the ground with at least one barefoot | | | | | | |

| | | | | | | | |
|---|---|---|---|---|---|---|---|
| 18 | List objects the baby puts in mouth or licks | | | | | | |
| | | **Number of Times Behavior was Observed in this Hour Block (TALLY)** | | | | | |
| | | **Hour 0 to 1** | | **Hour 1 to 2** | | **Hour 2 to 3** | |
| | **Other Behaviors** | **single event** | **sustained event for more than 5 seconds** | **single event** | **sustained event for more than 5 seconds** | **single event** | **sustained event for more than 5 seconds** |
| 19 | Object being put in mouth #1 | | | | | | |
| 20 | Object being put in mouth #2 | | | | | | |
| 21 | Object being put in mouth #3 | | | | | | |
| 22 | Object being put in mouth #4 | | | | | | |
| 23 | Object being put in mouth #5 | | | | | | |
| 24 | Other behavior #1 : | | | | | | |
| 25 | Other behavior #2 : | | | | | | |
| 26 | Other behavior #3 : | | | | | | |
| 27 | Other behavior #4 : | | | | | | |
| 28 | Other behavior #5 : | | | | | | |
